# The Influence of Coaxial Ultrasound on the Droplet Transfer of High Nitrogen Steel GMAW Process

**DOI:** 10.3390/ma17225509

**Published:** 2024-11-12

**Authors:** Jiawen Luo, Zhizheng He, Zeng Liu, Zihuan Hua, Bin Teng, Chenglei Fan

**Affiliations:** State Key Laboratory of Advanced Welding and Joining, Harbin Institute of Technology, Harbin 150001, China

**Keywords:** high nitrogen steel, ultrasonic-assisted GMAW, droplet transfer behavior, coaxial ultrasound

## Abstract

The nitrogen bubble bursting phenomenon during the welding process of high nitrogen steel (HNS) can lead to unstable droplet transfer and welding process, reducing the quality of weld formation. In this study, a novel approach, ultrasonic-assisted gas metal arc welding (U-GMAW), is proposed to suppress the escape of nitrogen gas during droplet transfer. This study investigates the influence of ultrasound on the metal transfer process during two distinct metal transfer modes: short-circuiting and droplet transfer. Ultrasound has a significant effect on the welding process; as ultrasonic power increases, both the arc length and droplet size decrease, while the droplet transfer frequency increases and the electrical signal stabilizes. Under the experimental conditions of this study, ultrasound has the most effective improvement on the metal transfer behavior when the ultrasonic power reaches 2 kW. Ultrasound enhances the stability of the droplet transfer process, making U-GMAW an effective and novel approach for controlling the droplet transfer behavior of high nitrogen steel.

## 1. Introduction

High nitrogen stainless steel (HNS) is a rapidly developing type of stainless steel in recent years. It utilizes nitrogen as the main austenite-forming element instead of nickel [1]. Nitrogen is a strong austenite-forming element. Dissolved nitrogen improves the strength and corrosion resistance of stainless steel [2]. HNS has promising applications in various fields, such as shipbuilding, electric power, and weapon manufacturing [3]. It is also used in the medical [4] and biological [5,6] fields due to its good compatibility with the human body. Recently, HNS welding wires have been utilized in the welding of HNS. However, the elevated nitrogen content presents challenges to fusion welding. During the Gas Metal Arc Welding (GMAW) process, the molten wire forms a droplet at the tip. Since the solubility of nitrogen in solids is much greater than that in liquids [7,8], nitrogen tends to accumulate in the molten droplet. When supersaturated nitrogen atoms aggregate to form nitrogen bubbles of a certain size, the droplet transfer becomes unstable, and the droplet may expand and splash, resulting in an unstable welding process and poor weld formation [9,10]. If the nitrogen bubbles trapped in the molten pool cannot escape before solidification, they will remain in the weld, forming gas pores that degrade joint quality. Furthermore, the loss of nitrogen content can alter the microstructure and negatively affect the mechanical properties of the joint [11,12]. Several studies have highlighted the instability of droplet transfer in the GMAW of HNS. Yang et al. [13] found the expansion, explosion, and splashing of large-sized HNS droplets captured by high-speed cameras. Liu et al. [14] found that with the increase in nitrogen content in the welding wire, the intensity of nitrogen gas escaping from the droplet gradually increases, leading to unstable arc and exploding droplets, which in turn cause spatter and smoke. Yang et al. [15] also reported that in the Wire Arc Additive Manufacturing (WAAM) process of high nitrogen austenitic stainless steel (HNASS), a sharp decrease in nitrogen solubility in the molten droplet caused violent explosions and splashing, leading to defects such as incomplete fusion, cracks, and inclusions.

A decrease in the dissolved nitrogen content in the weld joint adversely affects the mechanical properties, necessitating measures to prevent nitrogen loss. Many studies have addressed nitrogen loss by optimizing shielding gas composition and content or modifying the composition of welding wires. Cui et al. [16] found that the droplet transfer mode shifts to spray transfer when O_2_ is added to the shielding gas, improving weld formation and stability. The highest nitrogen content in the weld seam is achieved when using 89%Ar + 10%N_2_ + 1%O_2_ as the shielding gas. Liu et al. [14] designed three high nitrogen steel welding wires for GMAW. As the nitrogen content of the welding wire increased, nitrogen loss in the weld became more serious, and observation of the droplet transfer showed obvious growth and rupture upon droplet detachment. Ultrasonic vibration has also been shown to reduce nitrogen gas pores. Cui et al. [17] studied ultrasonic-assisted laser-arc hybrid welding of HNS. The porosity defects are suppressed under an ultrasonic power of 180 W, resulting in optimal mechanical properties. However, the nitrogen content decreases when the ultrasonic power exceeds a certain threshold, leading to a decrease in the hardness of the weld seam.

Research on the control of nitrogen content loss mainly focuses on the design of the components of shielding gas and welding wire to add nitrogen to the molten pool, while few studies on external auxiliary methods have been studied. Droplet transfer is a critical stage of GMAW, during which nitrogen bubbles are generated and ruptured. Droplet transfer behavior directly impacts welding quality, process stability, and efficiency of GMAW. Therefore, it is necessary to take measures to control the droplet transfer behavior of GMAW for high nitrogen steel, inhibit the growth of nitrogen bubbles, and thus reduce nitrogen loss. According to our previous research [18], ultrasound significantly compresses the arc, making it brighter compared to conventional GMAW. Additionally, the droplet transfer frequency and stability of the molten droplet increase under the influence of the acoustic radiation force.

This study aims to explore a novel approach to coaxial ultrasonic assistance for controlling the droplet transfer behavior in the GMAW process of HNS welding wire, with the goal of mitigating nitrogen loss. The ultrasonic probe vibrates coaxially with the welding wire, and the direction of ultrasonic vibration aligns with the transfer direction of the molten droplet. The influence of ultrasonic power on two typical droplet transfer modes was investigated by analyzing the droplet transfer behavior captured by a high-speed camera and the corresponding electrical signals. Subsequently, the mechanisms by which ultrasound influences these two droplet transfer modes were analyzed, providing a new method for controlling the droplet transfer behavior in HNS GMAW. By introducing ultrasonic assistance to the GMAW process, this study presents a unique perspective and practical solution for enhancing the stability of HNS droplet transfer behavior. It provides a strong guarantee for the safe and reliable operation of engineering welding structures and promotes the broader application of HNS in various industries.

## 2. Materials and Methods

The base material is 304 stainless steel, with dimensions of 130 mm × 50 mm × 7 mm, and the welding wire has a diameter of 1.2 mm, with nitrogen content of 0.35%, as shown in Table 1. The shielding gas composition is 98%Ar + 2%O_2_, and the gas flow rate is 20 L/min. The substrate surface was polished and cleaned to degrease with ethanol before deposition.

**Figure 1:**
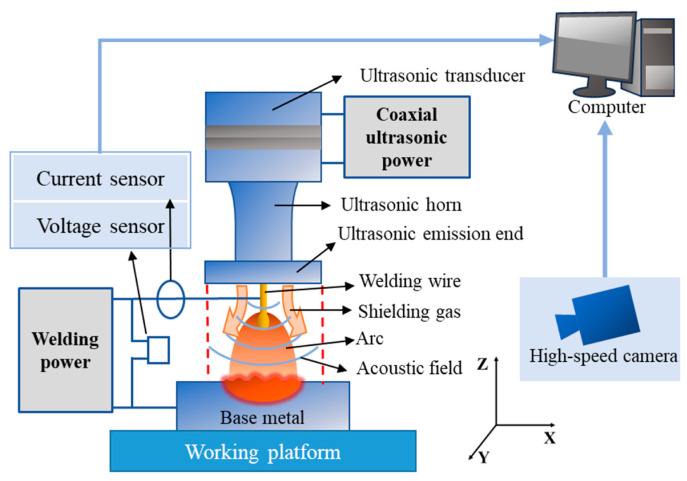
U-GMAW platform.

The ultrasonic-assisted GMAW (U-GMAW) platform consists of a welding system and a high-speed camera acquisition system, as illustrated in Figure 1. The ultrasonic transducer is coaxially integrated with the welding wire, and both the *z*-axis of the ultrasonic equipment and the GMAW welding gun can be independently adjusted. The maximum power output of the ultrasonic transducer is 2 kW. The experiments were conducted with the CLOOS QinTron welding machine (CLOOS, Haiger, Germany) operating on direct current. A high-speed camera captures images of the whole process of droplet formation and its transfer into the molten pool, with a photo resolution of 512 × 512 dpi, a capture frequency of 2000 fps, and exposure settings optimized for the specific experimental conditions. A filter with a wavelength of 808 nm and bandwidth of ±10 nm is placed in front of the camera to improve image quality. The high-speed video is subsequently played back in slow motion to analyze the details of the droplet transfer and analyze the droplet and arc behavior to determine the droplet transfer mode. The high-speed imaging system used is a PHANTOM v311 (Vision Research, Wayne, NJ, USA). Prior to welding, a copper plate is placed on the workbench to facilitate heat dissipation. The high nitrogen steel substrate is fixed on the copper plate and workbench using a fixture, allowing it to move along the slider at a controlled speed.

The distribution of droplet transfers of the HNS welding wire was analyzed. The welding parameters are presented in Table 2. Welding Voltages (*U*) of 22, 24.5, 26, 28.5 V, and 30 V, and Wire Feed Speeds (*WFS*) of 1.5, 3.5, 6, 8, and 10.5 m/min were selected. The high-speed camera captured the droplet transfer behavior of each experimental group and recorded their transfer modes. The main ultrasonic process parameters include the ultrasonic emission height (*H*, mm) and the ultrasonic power (*P*, kW). During the short circuit transfer, the ultrasonic emission height is set at the First Resonant Height (*H*_1_), where the ultrasonic effect is strongest. According to the preliminary experiment, *H*_1_ was measured as 14 mm. In the case of globular transfer, where the arc voltage is higher and the arc is longer, the ultrasonic emitter operates at the Second Resonant Height (*H*_2_) of 20 mm to avoid damage to the emitter and to maximize the ultrasonic radiation force.

## 3. Results

### 3.1. Distribution of Droplet Transfer Modes

**Figure 2:**
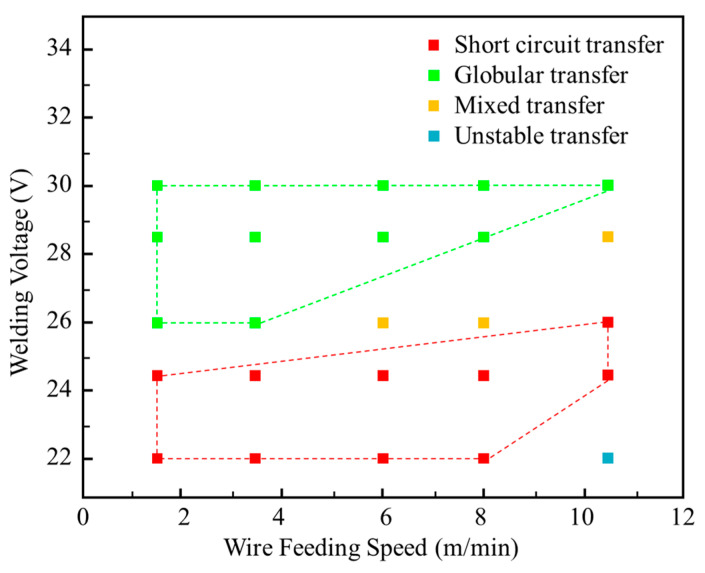
Parameter range of conventional GMAW droplet transfer modes.

**Figure 3:**
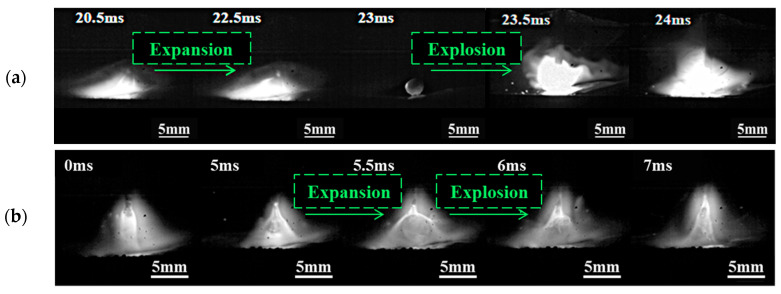
Droplet transfer process of short circuit transfer and globular transfer. (**a**) Short circuit transfer, *U* = 24.5 V, *WFS* = 6 mm/min; (**b**) Globular transfer, *U* = 28.5 V, *WFS* = 6 mm/min.

The distribution of droplet transfer modes under different welding parameters is presented in Figure 2. Four droplet transfer modes are observed: short circuit transfer (red area), globular transfer (green area), mixed transfer (orange area), and unstable transfer (blue area) among the 25 sets of parameters. The short circuit and globular transfer modes exhibit distinctly representative behaviors of droplet transfer, as shown in Figure 3. Therefore, ultrasound is applied in short circuit transfer and globular transfer modes for further investigation. In the conventional GMAW process, the expansion and explosion of droplets are notably evident. In Figure 3a, the droplet expands and then explodes upon contact with the molten pool during short circuit transfer. In Figure 3b, the molten droplet expands dramatically and then explodes, turning into a smaller and incomplete shape.

### 3.2. Effect of Ultrasound on Short Circuit Transfer Behavior

**Figure 4:**
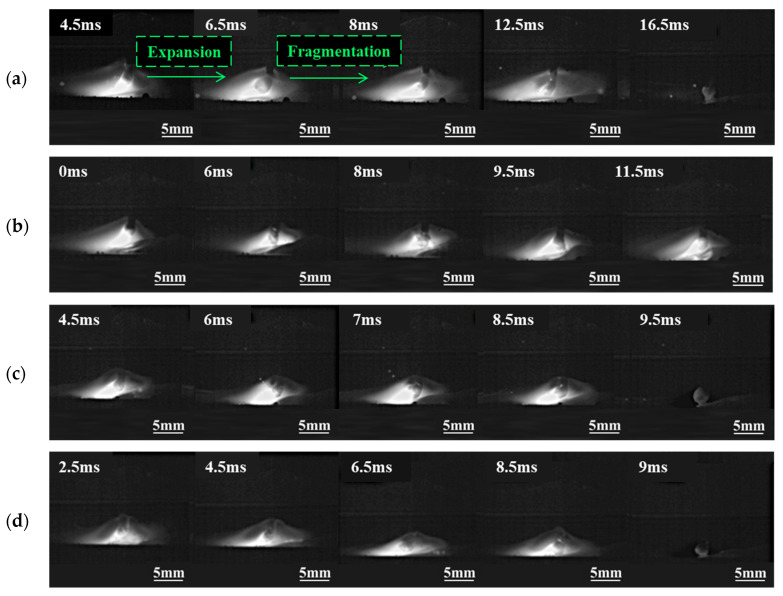
Influence of ultrasonic power on short circuit transfer. (**a**) 0.8 kW; (**b**) 1.2 kW; (**c**) 1.6 kW; (**d**) 2.0 kW.

The ultrasonic output power is set to 0.8, 1.2, 1.6, and 2.0 kW to investigate the effect of ultrasonic power on the short circuit transfer. Welding voltage is 24 V, *WFS* is 6 m/min. Figure 4. Illustrates the droplet transfer behavior captured by the high-speed camera. The arc is significantly compressed compared to conventional GMAW in Figure 3a. The droplet still notably expands at the end of the wire, and a certain amount of nitrogen gas escapes, leading to nitrogen loss. Then the droplet continues to expand until it contacts the weld pool, forming a short circuit transfer. When the ultrasonic output power is 1.2 kW, the large-sized expansion of droplets decreases, and the surface shape of the droplets shows a wavy fluctuation due to the pressure difference between the inside and outside of the droplet. Local fragmentation and escape of a small amount of nitrogen gas occur in some areas occasionally, mainly due to insufficient surface tension and ultrasonic radiation force to balance the internal pressure exerted by nitrogen gas within the droplet. At other times, intense expansion can cause the rupture of molten droplets. When the ultrasonic output power increases to 1.6 kW and 2.0 kW, the droplet transfer phenomenon is similar to that at 1.2 kW, although the ultrasonic radiation force increases, there are still instances of droplet fragmentation and a certain degree of expansion and explosion, but the frequency Is relatively less. Increasing the ultrasonic power also Increases transfer frequency, causing the droplets to contact the weld pool before experiencing large-sized expansion, leading to reduced nitrogen escape.

**Figure 5:**
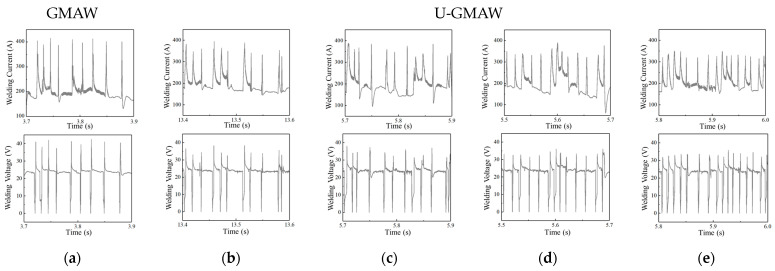
Current and voltage waveforms of conventional GMAW and U-GMAW. (**a**) Conventional GMAW; (**b**) 0.8 kW; (**c**) 1.2 kW; (**d**) 1.6 kW; (**e**) 2.0 kW.

The electric signals of the droplet transfer process were collected under conventional GMAW and different ultrasonic power levels, as shown in Figure 5. Under conventional GMAW conditions, the droplet transfer frequency is low, with a peak current reaching 400 A and a peak voltage of 40 V. When an ultrasonic power of 0.8 kW is applied, the number of wave peaks increases, and the peak current decreases to approximately 370 A, and the peak voltage decreases to around 37 V. As the ultrasonic power is increased to 1.2 kW and 1.6 kW, the number of wave peaks increases, and the peak current and voltage further decrease. At 2.0 kW ultrasonic power, compared to the previous conditions, the number of current and voltage wave peaks is the highest and the lowest, while the peak current drops to around 350 A and the peak voltage decreases to around 35 V. Analysis indicates that under conventional GMAW conditions, the re-ignition time of the arc after the droplet short circuit is long and the droplet transfer is unstable. However, with ultrasonic assistance, an increase in ultrasonic output power accelerates droplet transfer frequency, while the peak current and voltage decrease during short circuit transfer, indicating a shortened re-ignition time after the short circuit transfer and a more stable droplet transfer process.

**Figure 6:**
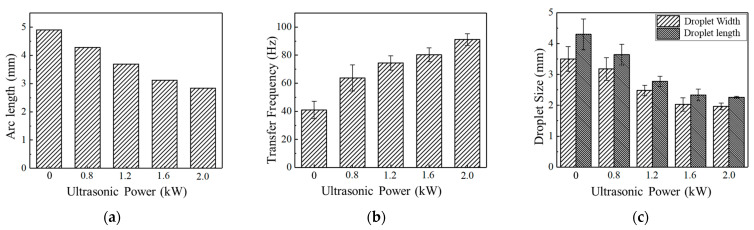
Short circuit transfer characteristic parameters under different ultrasonic powers. (**a**) Arc length; (**b**) Transfer frequency; (**c**) Droplet size.

Statistical analysis of droplet characteristic parameters at different ultrasonic power levels is shown in Figure 6. As the ultrasonic output power increases, the arc length gradually decreases, reaching a minimum at 2.0 kW. With the increase of ultrasonic power, the short circuit transfer frequency increases, and the droplet size decreases, exhibiting a reduction in numerical dispersion. This suggests that at higher ultrasonic power, the expansion of large droplets is suppressed, and the short circuit transfer frequency becomes more stable.

### 3.3. Effect of Ultrasound on Globular Transfer Behavior

**Figure 7:**
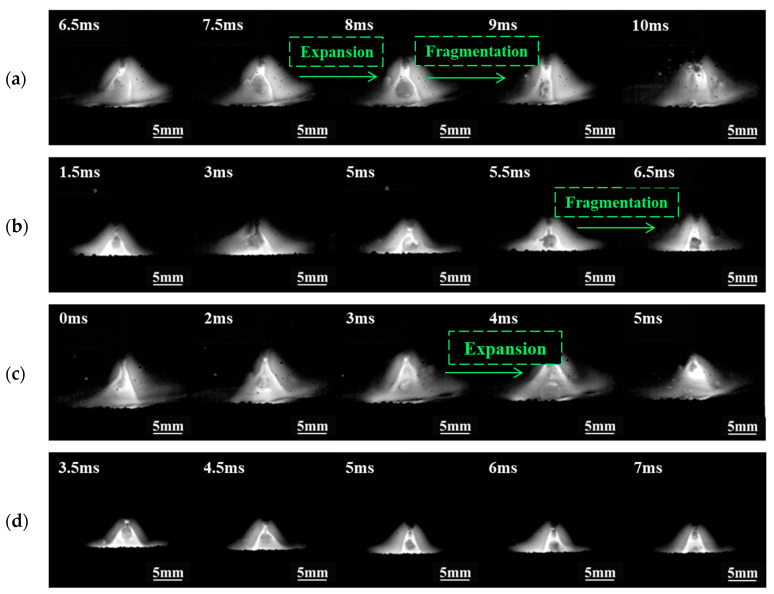
Influence of ultrasonic power on globular transfer. (**a**) 0.8 kW; (**b**) 1.2 kW; (**c**) 1.6 kW; (**d**) 2.0 kW.

Figure 7 illustrates the globular transfer behavior under different ultrasonic power levels with a *WFS* of 6 m/min. At lower ultrasonic powers (0.8 kW and 1.2 kW), the arc length is significantly reduced compared to conventional GMAW in Figure 3b. However, droplets still expand and explode, suggesting insufficiency in ultrasonic radiation force. When the ultrasonic output power reaches 1.6 kW and 2.0 kW, the arc length decreases further, with a marked reduction in large-sized expansion. Nonetheless, some degree of expansion persists, and irregular surface shapes are noted in the droplets. Local fragmentation of droplets is observed, yet nitrogen emissions from fragmentation are lower than those resulting from large-scale bursting under conventional GMAW conditions. This indicates that ultrasonic assistance, to some extent, reduces nitrogen escape during droplet transfer in high nitrogen steel welding, thereby reducing nitrogen loss.

**Figure 8:**
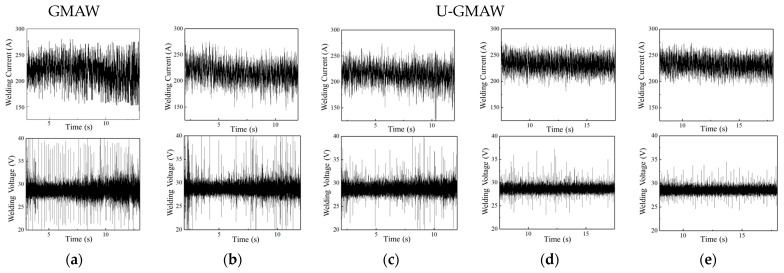
Current and voltage waveforms of conventional GMAW and U-GMAW. (**a**) conventional GMAW; (**b**) 0.8 kW; (**c**) 1.2 kW; (**d**) 1.6 kW; (**e**) 2.0 kW.

The electrical signals at different ultrasonic power were collected, as shown in Figure 8. The current fluctuation range is 170 A to 270 A, and the welding voltage fluctuation range is 20 V to 40 V under conventional GMAW. The large fluctuations in the current and voltage values indicate the instability of the arc and the droplet transfer during globular transfer in GMAW. With the assistance of 0.8 kW ultrasound, the current fluctuation range narrows to 180 A to 250 A, indicating an improvement in the stability of the droplet transfer process. As the ultrasonic output power increases, the fluctuation range of the current and voltage values progressively decreases, thereby enhancing the stability of droplet transfer while mitigating the expansion and bursting of large droplets.

**Figure 9:**
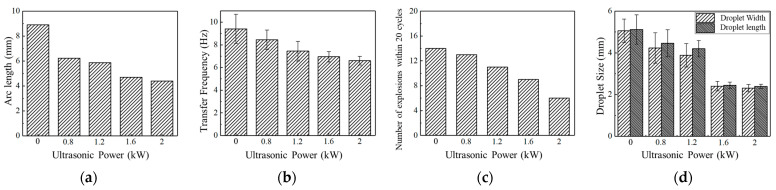
Globular transfer characteristic parameters at different ultrasonic powers. (**a**) Arc length; (**b**) Transfer frequency; (**c**) Number of explosions within 20 cycles; (**d**) Droplet size.

Figure 9 illustrates the droplet transfer characteristic parameters under different ultrasonic power levels. As ultrasonic power increases, the ultrasonic radiation force increases, causing the arc length to gradually decrease, accompanied by a reduction in droplet transfer period, bursting frequency, and droplet size, reaching the minimum values at an ultrasonic output power of 2.0 kW. This indicates the effective inhibition of large-scale droplet expansion under high ultrasonic power conditions. Regarding numerical dispersion, the magnitude of numerical variations decreases as ultrasonic output power increases. A sharp reduction in numerical dispersion is observed under ultrasonic power of 1.2 kW and 1.6 kW, where most droplets exhibit stable transfer with relatively minor size expansion. The most stable droplet transfer is achieved under 2.0 kW.

## 4. Discussion

Based on the arc constriction and droplet size reduction induced by ultrasonic radiation force, coaxial ultrasonic assistance presents a promising approach to controlling droplet transfer behavior in GMAW of high nitrogen steel. The discussion on the mechanism of droplet transfer in ultrasonic-assisted GMAW is as follows.

### 4.1. Mechanism of Ultrasound on Short Circuit Transfer

**Figure 10:**
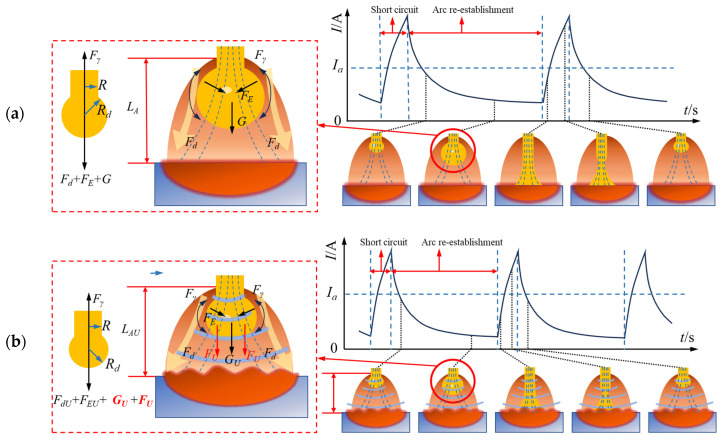
Schematic of the short circuit transfer process of GMAW and U-GMAW. (**a**) GMAW; (**b**) U-GMAW.

The process diagram of short circuit transfer behavior in GMAW and U-GMAW is presented in Figure 10. During the arcing phase, molten droplets form at the tip of the welding wire. As the droplets grow, a short circuit occurs when they contact with the weld pool. Under the combined effects of electromagnetic forces and surface tension, necking occurs, separating the molten droplet from the welding wire. The arc then re-ignites to initiate a new transfer cycle. While the short circuit transfer process in U-GMAW is similar to conventional GMAW, it features an increased transfer frequency and decreased arc length.

During conventional GMAW, as illustrated in Figure 10a, the molten droplets at the tip of the welding wire are subjected to electromagnetic force *F_m_*, plasma flow force *F_d_*, gravity *G*, and surface tension *F_γ_*. The combined forces of *F_m_*, *F_d_*, and *G* act downward, promoting droplet transfer. When the droplet has not yet detached from the welding wire, *F_γ_* prevents the droplet from falling, as expressed by Equation (1) (where *R* represents welding wire diameter and *γ* represents surface tension coefficient). Notably, *F_γ_* is not significantly influenced by ultrasound.

(1) $$F_{\gamma }=2\pi R\gamma$$

Equation (2) is the expression for the electromagnetic force *F_m_* acting on a unit volume, where $\overset{⃑}{J}$ represents the current density, and $\overset{⃑}{B}$ represents the magnetic field line density.

(2) $$F_{m}=\overset{⃑}{J}\times \overset{⃑}{B}$$

During the welding process, droplets serve as channels for current flow and are also subjected to electromagnetic forces. According to the high-speed camera results, under the experimental conditions of this study, the arc nearly envelops the molten droplets, causing *F_m_* to promote the downward transfer of the molten droplets [19], as expressed by Equation (3).

(3) $$F_{m}=\frac{\mu_{0}I^{2}}{4\pi }\left(\mathrm{In}\frac{R_{d}\sin\theta }{R}-\frac{1}{4}-\frac{1}{1-\cos\theta }+\frac{2}{{\left(1-\cos\theta \right)}^{2}}\mathrm{In}\frac{2}{1-\cos\theta }\right)$$

where *μ*_0_ represents the vacuum permeability, *I* represents the welding current, *R_d_* represents the droplet diameter, and *θ* is the angle between the line connecting the droplet center and the contact point of the arc on the droplet and the direction of gravity. In this study, the molten droplets are enveloped by the arc, making *θ* greater than 60° degrees. The coefficient in parentheses of Equation (3) is close to 1, and the electromagnetic force can be expressed in Equation (4). The electromagnetic force in this study is mainly affected by welding current.

(4) $$F_{m}=\frac{\mu_{0}I^{2}}{4\pi }$$

Plasma flow force *F_d_*, detailed in Equation (5), also has an effect on molten droplets [20]:

(5) $$F_{d}=0.5C_{d}\rho_{g}v_{g}^{2}\pi R_{d}^{2}\left(1-\frac{R^{2}}{2R_{d}^{2}}\right)$$

where *C_d_* represents the plasma flow coefficient, related to the droplet diameter *D_d_* and viscosity *μ*, *ρ_g_* represents the gas density, and *v_g_* represents the gas flow rate.

The droplet gravity is expressed in Equation (6):

(6) G=ρVg

where *ρ* represents the density of molten droplets, *V* represents droplet volume, and *g* represents gravitational acceleration.

During U-GMAW, the ultrasonic device emits ultrasound in a downward direction, generating a standing wave sound field and exerting acoustic radiation pressure on the molten droplets. The ultrasonic radiation force *F_U_* is expressed in Equation (7), where *ρ_m_* represents the density of the sound propagation medium, *A* represents the velocity amplitude of the incident wave, *k* represents the number of waves, *R_d_* represents the droplet radius m, and *λ_p_* represents the ratio of medium density to droplet density [18].

(7) $$F_{U}=\frac{1}{3}\pi \rho_{m}{\left|A\right|}^{2}{\left(kR_{d}\right)}^{3}\sin\left(2\mathrm{kd}\right)\frac{5-2\lambda_{p}}{2+\lambda_{p}}$$

Based on the research results mentioned above, it is evident that the introduction of ultrasound compresses the molten droplets, reducing their volume and consequently lowering the gravitational force (*G_U_* < *G*), which impedes droplets from making contact with the weld pool below. Reduced droplet size also leads to a decrease in *F_dU_* (*F_dU_* < *F_d_*). The electromagnetic force will slightly decrease (*F_mU_* < *F_m_*), as ultrasound will cause a decrease in the peak current of short circuit transfer. However, the downward pressure exerted by the ultrasound field (*F_U_*) compensates for the reduction in *G_U_*, *F_dU_*, and *F_mU_*. The force exerted by ultrasound on molten droplets is the main reason for promoting droplet transfer.

The ultrasound significantly compresses the arc, reducing its length and facilitating easier contact between the molten droplets and the weld pool, thereby promoting short circuit transfer. Additionally, ultrasound radiation forces oscillate the molten weld pool, causing surface fluctuations that facilitate faster contact between droplets and the weld pool, thereby accelerating the transfer frequency. Consequently, ultrasound promotes short circuit transfer, allowing the droplets to make contact with the weld pool and undergo short circuit transfer even when the nitrogen bubble aggregation in the droplets is minimal, thus reducing nitrogen loss.

### 4.2. Mechanism of Ultrasound on Globular Transfer

**Figure 11:**
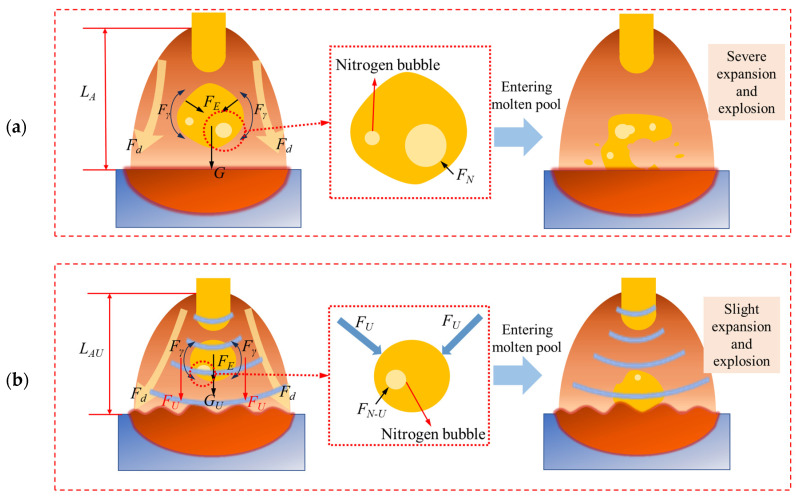
Schematic of the globular transfer process of GMAW and U-GMAW. (**a**) GMAW; (**b**) U-GMAW.

The schematic depiction of the globular transfer behavior of GMAW and U-GMAW is presented in Figure 11. The droplet transfer process differs from that of short circuit transfer, where the molten droplet detaches from the welding wire tip and then enters the weld pool. Similar to short circuit transfer, ultrasound radiation force compresses the droplet, reducing nitrogen accumulation.

Additionally, ultrasound has an inhibitory effect on the formation of nitrogen bubbles. Research has shown that the factors affecting the solubility of nitrogen in steel mainly include nitrogen partial pressure, temperature, and alloy composition [21]. During the droplet transfer process, the alloy composition of high nitrogen steel droplets remains unchanged. Given the rapid droplet transfer process, and assuming that the temperature of the droplets remains constant from the welding wire tip to entering the molten pool, nitrogen partial pressure is the primary factor affecting the solubility of nitrogen in molten droplets. Nitrogen dissolved in molten iron exists in the form of nitrogen ions, but nitrogen in molten steel often exists in the form of nitrogen atoms [22]. The reaction of nitrogen dissolution in liquid steel is:

(8) $$\frac{1}{2}N_{2}\left(g\right)=\left[N\right]$$

*K*_N_ is the equilibrium constant of nitrogen dissolution reaction, as measured in Equation (9) [23], where *a*_N_ and *f*_N_ are the nitrogen activity and Henrian activity coefficients of the assumed 1% N alloy melt as reference states, [% N] represents the mass percentage of dissolved nitrogen, $P_{N_{2}}$ is the partial pressure of nitrogen, and *P*^0^ is a standard atmospheric pressure of 1.01325 × 10^5^ Pa.

(9) $$K_{N}=\frac{a_{N}}{\sqrt{P_{N_{2}}/P^{0}}}=\frac{f_{N}\left[\%N\right]}{\sqrt{P_{N_{2}}/P^{0}}}$$

Taking the logarithm of both sides of Equation (9) yields Equation (10):

(10) $$\mathrm{lg}\left[\%N\right]=\frac{1}{2}\mathrm{lg}\left(P_{N_{2}}/P^{0}\right)+\mathrm{lg}K_{N}-\mathrm{lg}f_{N}$$

The *K*_N_ of steel can be expressed as Equation (11) [24], and *K*_N_ can be considered a constant at a constant temperature.

(11) $$\mathrm{lg}K_{N}=-\frac{188}{T}-1.17$$

*f*_N_ is mainly affected by temperature and alloy composition [25]. During the droplet transfer process, the composition and temperature changes of the droplets are negligible, and *f*_N_ is considered a constant value. Thus, the solubility of nitrogen is mainly influenced by the partial pressure of nitrogen. The dissolved nitrogen concentration in the droplet during the droplet transfer of GMAW and U-GMAW is primarily determined by the nitrogen partial pressure. According to Equation (11), [% N] and ($P_{N_{2}}/P^{0}$)^1/2^ are approximately proportional, meaning the solubility of nitrogen increases as the nitrogen partial pressure increases. The ultrasonic radiation applied to droplets increases the pressure ($P_{N_{2}-U}\;>P_{N_{2}}$) on nitrogen bubbles in the droplets, resulting in an increase in nitrogen solubility. This effect mitigates the formation of nitrogen bubbles and subsequently reduces the tendency for nitrogen escape. Furthermore, as ultrasonic power intensifies, the radiation force exerted by ultrasound increases, further reducing the propensity of nitrogen escape.

## 5. Conclusions

The study investigated the droplet transfer behavior of wires with nitrogen contents of 0.35% in ultrasonic-assisted GMAW (U-GMAW), focusing on short circuit transfer and globular transfer. The influence of ultrasonic output power on droplet transfer behavior was investigated, and the underlying mechanisms were analyzed. The main conclusions are summarized as follows:

1. Ultrasonic power has a significant influence on the droplet transfer process of HNS welding wire. Ultrasonic assistance improves the stability of droplet transfer: as ultrasonic power increases, the arc is further compressed, leading to a reduction in arc length. Droplet expansion is effectively inhibited under both short circuit transfer and globular transfer modes.
2. The influence of ultrasonic assistance on welding electrical signals was studied. It was found that the droplet transfer frequency is lower during conventional GMAW short circuit transfer, with relatively high peak current and voltage under both transfer modes. As the ultrasonic power increases, the short circuit transfer frequency increases, and the peak current and voltage under both modes decrease, making the droplet transfer process more stable.
3. The behavior mechanisms of short circuit transfer and globular transfer in U-GMAW were analyzed. The ultrasonic radiation force compresses the arc length, accelerates droplet transfer frequency, and suppresses droplet expansion, thereby reducing nitrogen accumulation within the droplets. Consequently, more nitrogen elements can be transferred into the molten pool during welding with HNS welding wires, leading to a reduction in nitrogen loss.

## Figures and Tables

**Table 1:** Composition of High Nitrogen Steel Welding Wire (wt.%).

Element	C	Si	Mn	Cr	Ni	Mo	N
Content	0.071	0.832	8.84	22.26	6.54	0.27	0.35

**Table 2:** Welding parameters.

Welding Current (*I*)/A	Welding Voltage (*U*)/V	Wire Feeding Speed (*WFS*)/(m·min^−1^)
120~350	20~32	1.5~12

## Data Availability

The raw data supporting the conclusions of this article will be made available by the authors on request.
